# Is Wetter Better? An Evaluation of Over-the-Counter Personal Lubricants for Safety and Anti-HIV-1 Activity

**DOI:** 10.1371/journal.pone.0048328

**Published:** 2012-11-07

**Authors:** Charlene S. Dezzutti, Elizabeth R. Brown, Bernard Moncla, Julie Russo, Marilyn Cost, Lin Wang, Kevin Uranker, Ratiya P. Kunjara Na Ayudhya, Kara Pryke, Jim Pickett, Marc-André LeBlanc, Lisa C. Rohan

**Affiliations:** 1 Magee-Womens Research Institute, University of Pittsburgh, Pittsburgh, Pennsylvania, United States of America; 2 University of Pittsburgh, Pittsburgh, Pennsylvania, United States of America; 3 Statistical Center for HIV/AIDS Research and Prevention, Fred Hutchinson Cancer Research Center, Seattle, Washington, United States of America; 4 International Rectal Microbicide Advocates, Chicago, Illinois, United States of America; Burnet Institute, Australia

## Abstract

Because lubricants may decrease trauma during coitus, it is hypothesized that they could aid in the prevention of HIV acquisition. Therefore, safety and anti-HIV-1 activity of over-the-counter (OTC) aqueous- (n = 10), lipid- (n = 2), and silicone-based (n = 2) products were tested. The rheological properties of the lipid-based lubricants precluded testing with the exception of explant safety testing. Six aqueous-based gels were hyperosmolar, two were nearly iso-osmolar, and two were hypo-osmolar. Evaluation of the panel of products showed Gynol II (a spermicidal gel containing 2% nonoxynol-9), KY Jelly, and Replens were toxic to *Lactobacillus*. Two nearly iso-osmolar aqueous- and both silicone-based gels were not toxic toward epithelial cell lines or ectocervical or colorectal explant tissues. Hyperosmolar lubricants demonstrated reduction of tissue viability and epithelial fracture/sloughing while the nearly iso-osmolar and silicon-based lubricants showed no significant changes in tissue viability or epithelial modifications. While most of the lubricants had no measurable anti-HIV-1 activity, three lubricants which retained cell viability did demonstrate modest anti-HIV-1 activity *in vitro*. To determine if this would result in protection of mucosal tissue or conversely determine if the epithelial damage associated with the hyperosmolar lubricants increased HIV-1 infection *ex vivo*, ectocervical tissue was exposed to selected lubricants and then challenged with HIV-1. None of the lubricants that had a moderate to high therapeutic index protected the mucosal tissue. These results show hyperosmolar lubricant gels were associated with cellular toxicity and epithelial damage while showing no anti-viral activity. The two iso-osmolar lubricants, Good Clean Love and PRÉ, and both silicone-based lubricants, Female Condom 2 lubricant and Wet Platinum, were the safest in our testing algorithm.

## Introduction

Microbicides are topical products which would be applied vaginally or rectally prior to coitus to prevent the sexual transmission of HIV-1. While film, suppository, cream, douche and intravaginal ring delivery devices are being developed, the most common delivery vehicle being evaluated for microbicides is gels. Because of their availability, over-the-counter (OTC) lubricants and vaginal moisturizers have been used as surrogates for microbicide gels in acceptability studies [1], [2], [3], [4] or as “placebos” for microbicides in safety studies [5], [6], [7], [8], [9], [10]. Conversely, microbicide studies have reported the lubricant properties of the gel products were appreciated by the participants; 25% to 70% of them thought the gels enhanced their sexual pleasure [5], [11], [12], [13]. It is anticipated that persons currently using lubricant products will incorporate microbicides into their sexual routine.

Globally, the majority of new HIV-1 infections occur via sexual transmission. In many parts of the world, including sub-Saharan Africa, there is limited or no access to OTC lubricant products. Because microtrauma that can occur during sex [14], [15], [16] could create portals of entry, there is an assumption that lubricant use could help reduce the risk of sexual transmission of HIV-1 [14]. The risk of HIV-1 acquisition during coitus is greatest for those persons engaging in receptive anal intercourse without using a condom [17], [18]. A recent study showed that men who have sex with men (MSM) and engage in anal intercourse frequently use OTC lubricant products without condoms [19]. However, little information has been documented regarding safety of OTC lubricants. Categorized as a “medical device” by the U.S. FDA, the components of lubricant products must be constituted from ingredients listed from several sources including the “GRAS” or generally recognized as safe list, FDA inactive ingredient list, code of Federal Regulations [20], or the Handbook of Pharmaceutical Excipients. Generally, limited safety testing has been done on these products.

Because OTC lubricant products are utilized in microbicide trials and are used most frequently by persons engaging in coitus with the highest risk of HIV-1 acquisition, it is important to know their impact on the tissues where they will be applied, which are the gastrointestinal and female genital tracts. Several products representing aqueous-, lipid-, and silicone-based gels were evaluated for their safety and anti-HIV-1 activity using our pre-clinical testing algorithm which is the same one used to evaluate formulated microbicides [21]. Knowing which products are safest for vaginal and rectal use is critical for consumers’ health and interpretation of clinical trial results.

## Materials and Methods

### Products Evaluated

OTC lubricant products were purchased from www.cheaplubes.com. Included in alphabetical order were Astroglide, Boy Butter H_2_O, Boy Butter Original, Elbow Grease, Good Clean Love, ID Glide (ultra long-lasting), KY Jelly, Slippery Stuff, Sliquid Organic, and Wet Platinum. Additional products tested were PRÉ, Replens, and Gynol II purchased from www.drugstore.com. Gynol II contains 2% nonoxynol 9 (N9) and was used as a toxicity control in the appropriate experiments. Female Condom 2 (FC 2) lubricant was supplied by the Female Health Company (London England), the manufacturer of the Female Condom. For each of the lubricant products, the formulation without additional flavors, scents, or other enhancements (e.g. “warming gel”) was used.

### Human Tissue and Epithelial Cell Lines

Normal human ectocervical and colorectal tissues were acquired from pre-menopausal women undergoing hysterectomy or persons undergoing colorectal surgery for non-inflammatory conditions, respectively. All tissue was obtained through IRB approved protocols (#0503103 and #0602024) at the University of Pittsburgh collected through an Honest Broker de-linking patient identifiers to the investigators. Tissue was placed in L-15 medium supplemented with 10% fetal bovine serum (FBS), 100 µg/ml streptomycin, 100 U/ml penicillin, and 2.5 µg/ml Amphotericin B and transported to the laboratory on ice and processed within 4 hours of surgery.

Epithelial cell lines were obtained from the American Type Culture Collection (Manassas, VA). Unless otherwise stated, culture reagents were purchased from Hyclone (Logan, UT). Caco-2 cells, a colorectal epithelial cell line [22], were grown in MEM alpha medium supplemented with 20% heat-inactivated FBS (Gemini Bio-products, West Sacramento, CA), 100 µg/ml streptomycin, 100 U/ml penicillin, and 100 mM L-glutamine. HEC-1-A cells, an endometrial epithelial cell line [23], were grown in McCoy’s 5A medium supplemented with 10% FBS, 100 µg/ml streptomycin, 100 U/ml penicillin, and 100 mM L-glutamine. TZM-bl cells, a HeLa epithelial cell line stably transfected with an HIV-1 long terminal repeat linked to a β-galactosidase and luciferase genes [24], were grown in DMEM medium supplemented with 10% FBS, 100 µg/ml streptomycin, 100 U/ml penicillin, and 100 mM L-glutamine.

### Physicochemical Testing

The major physicochemical parameters typically evaluated for aqueous semi-solids include viscosity, osmolality, and pH. Viscosity was determined using the CP51 spindle on a cone/plate Brookfield Model HADVIII viscometer (Brookfield Eng. Lab., Inc., Middleboro, MA). The sample was placed in the sample cup of the instrument and allowed to equilibrate to 37°C for 10 min. Viscosity was measured using a program where speed was increased from 0.2 to 30 rpm and subsequently decreased to 0.2 rpm. To compare data across samples, viscosity values acquired at 10 rpm at 25 and 37°C (shear rate = 38.4 sec-1) were used in the analysis. Osmolality was determined using a Vapor Pressure 5520 Osmometer (Wescor, Inc., Logan, UT) calibrated with Opti-mole 290 and 1000 mmol/kg osmolality standards. Products with high osmolality were diluted two to three times (w/w) with DI water before testing since the Wescor osmometer has an upper limit for use at 3200 mmol/kg. pH was determined using an Accumet AR20 pH meter (Fisher) with an a flat surface pH electrode (Beckman Coulter Futura Flat Bulk Combination pH electrode) calibrated using three points, pH 4.0, 7.0 and 10.0.

### Safety Testing

#### Normal vaginal flora testing

One strain of *L. crispatus* (ATCC 33197) and two strains of *L. jensenii* (ATCC 25258 and LBP 28Ab) were obtained from the American Type Culture Collection (Manassas, VA.). Minimum cidal concentrations, the concentration required to reduce the viability of a culture by 99.99%, were determined as previously described [25], [26]. Briefly, bacterial suspensions were prepared by selecting isolated colonies from fresh overnight culture plates and suspending the test organisms in saline to a density of 2×10^8^ bacteria/ml (McFarland standard of 2.0). OTC lubricant products were mixed with an equal volume of the bacterial suspensions at room temperature and plated for colony forming units (CFUs). The number of CFUs was taken from the dilution plate containing 50 to 300 colonies. All reported values represent the average of triplicate experiments. Samples were taken at time 0 and after 30 min of exposure. Samples yielding 10 or fewer CFUs (representing a 99.99% kill) were considered sensitive to killing. All results were compared to the control which was identical but lacking the OTC lubricant products.

#### Epithelial cell lines testing

Dilutions (1∶10, 1∶100, 1∶500, 1∶1000, 1∶5000, and 1∶10,000) were made of the aqueous gels in the appropriate cell culture medium to test for cell viability. FC2 lubricant and Wet Platinum were used neat. For cell viability, Caco-2, HEC-1-A, or TZM-bl cell lines were plated in triplicate in a 96-well plate for each treatment. Dilutions of gels made in the appropriate medium were added to the appropriate wells. FC2 lubricant (100 µl) and Wet Platinum (100 µl) were applied for 15, 30 or 60 min and then 100 µl of appropriate medium was added. Control wells with no treatment (cells only) were included for background luminescence. The plate was cultured for 24 h at 37°C/5% CO_2_ and then washed twice with Hank’s Balance Salt Solution (Ca++/Mg++-free) (HBSS). After the last wash, CellTiter-Glo™ (Promega Corp., Madison, WI) was added to all the wells and luminescence was measured using a Beckman DTX 880 plate reader. Viability was determined based on deviations from the cell only control and presented as % viability ± standard deviation.

To determine the effect of the aqueous gels on epithelial integrity, the TER was measured as described previously [27]. HEC-1-A or Caco-2 cells were grown on transwell supports until a polarized monolayer developed as determined by a plateau in resistance as measured by a MilliCell-ERS resistance system (Millipore, Billerica, MA). At that time, a 1∶10 dilution of each aqueous lubricant was added to the apical surface of the monolayer and resistance readings were measured over a 24 h period. As controls, wells with no cells (background resistance) and wells with cells alone were used. The epithelial resistance was expressed as (resistance of the treated wells – resistance of the no cell wells) ÷ (resistance of the cells alone wells – resistance of the no cell wells) = Ω×cm^2^. FC2 lubricant or Wet Platinum was applied (100 µl each) directly onto confluent epithelial cells for up to 60 min and then 500 µl of medium containing fluorescent microspheres (Microgenics Corp., Fremont, CA) was applied apically. Basolateral aliquots were taken over 24 h and stored in a black 96-well plate (Greiner Bio-One, Monroe, NC) wrapped in foil at 4°C. After the experiment was complete, the plate was read on the DTX880 plate reader. Epithelial integrity was measured as a % of microspheres that passed through the monolayer over time in the treated wells compared to the wells with no cells (100% transmission).

#### Explant culture testing

Polarized ectocervical and colorectal explant cultures were set-up as previously described [28], [29]. The explants were prepared on day of surgery in duplicate. To ensure even spread and to allow them to be mixed with HIV-1 for the susceptibility testing (below), a 1∶5 dilution of aqueous lubricant gels was applied to the apical side of the explants for 18 h. FC 2 lubricant and Wet Platinum were applied undiluted. As controls, untreated explants or a 1∶5 dilution of N9 gel was applied apically. The next day, explants were washed and viability was evaluated using the MTT [1-(4,5-dimethylthiazol-2-yl)-3,5-diphenylformazan] assay and histology [28], [29].

### Anti-HIV-1 Testing

To determine if the lubricants have any anti-viral capacity, the dilutions of the aqueous lubricant were applied to TZM-bl cells to determine the 50% cytotoxic concentration and the 50% efficacious dose to calculate a therapeutic index (TI = CC_50_ ÷ ED_50_). The toxicity was determined as described above. Using the same dilutions of each aqueous lubricant, 3000 TCID_50_ of HIV-1_BaL_ with 4 µg/ml of DEAE-Dextran were added to each well. FC2 lubricant or Wet Platinum (100 µl) was applied for up to 60 min and then 100 µl of medium with HIV-1 and DEAE-Dextran was added. Controls included medium alone (background luminescence) and HIV-1 only (maximum luminescence). All treatments were tested in triplicate. The plate was cultured for 48 h at 37°C/5% CO_2_ and then washed twice with HBSS. After the last wash, BrightGlo™ (Promega Corp., Madison, WI) was added to all the wells per the manufacturer’s instructions and luminescence was measured using a Beckman DTX 880 plate reader.

To determine if the lubricants increase susceptibility to HIV-1 infection, representative gels (Astroglide, Good Clean Love, KY Jelly, PRÉ, Replens, Sliquid Organic, and Wet Platinum) were applied to the apical surface of non-activated, polarized ectocervical explants in duplicate (set-up as described above). Explants with medium only were used as infection controls. After 18 h, the explants were washed with HBSS to remove residual gel. On two additional explants, 0.1% EDTA was added to the apical surface and cultured for 2 h. After washing, 5×10^3^ TCID_50_ of HIV-1_BaL_ was applied to the apical surface of all explants for 18 h and then washed with HBSS. Fresh medium was applied to the basolateral compartment. Every 3 to 4 days medium was harvested and stored at −80°C, and fresh medium reapplied for up to 21 days. The stored supernatant was tested for HIV-1 replication using the p24gag ELISA (Perkin-Elmer Life and Analytical Sciences, Inc., Waltham, MA). Immunohistochemistry (IHC) was performed for HIV-1 infected cells by staining for p24gag [29].

### Statistical Analysis

To assess differences in percent viability and percent transmission between experimental and control groups, an ANOVA model was fit with log-transformed outcomes controlling for repeated measures. Exponentiated coefficients from the regression model were then used as consistent estimators of percent viability and percent transmission and were tested to assess difference from 0 with F-tests with Bonferroni adjustments for multiple comparisons within the assay.

To assess the difference between the gel and control TER, an ANOVA model was fit with the difference between the gel and control TER measures as the outcome adjusting for measurement time and replication and the -1 day measurement. The difference at each time point was estimated and tested using an F-test to determine whether it differs from the baseline pre-exposure difference between the groups with Bonferroni adjustments for multiple comparisons.

Linear mixed effects models with random intercepts were used to assess the difference in p24 between lubricant-treated and control tissues over time. T-tests with Bonferroni adjustments for multiple comparisons were used to test for differences over time.

## Results

### Lubricant Formulation Testing

To better understand how the OTC lubricant products may perform in our experiments, basic formulation characterizations such as pH, osmolality, and viscosity testing were done (Table 1) [30]. The pH and osmolality were determined for the 10 aqueous-based gels tested. Seven of them were acidic. The other three had a neutral pH. Boy Butter H_2_O, a lipid-based gel that is water soluble, had a neutral pH. Good Clean Love and PRÉ were within 2-fold of iso-osmolar. Sliquid Organic was 2.7-fold and Slippery Stuff was 11-fold below iso-osmolar (hypo-osmolar). The remaining aqueous-based products and Boy Butter H_2_O had osmolalities ranging from 4.8- to 13.3-fold above iso-osmolar. To note, Astroglide was 21.1-fold higher than iso-osmolar. The viscosities ranged from 145 cps (very thin) for Wet Platinum to 22783 cps (very thick) for Gynol II at room temperature. Elbow Grease, Gynol II, ID Glide, PRÉ, and the two lipid-based products viscosities decreased by 20% to 46% when the temperature was increased to 37°C which indicates the products became thinner and more spreadable at body temperature.

**Table 1:** Physical characteristics of the over-the-counter lubricants tested.

Lubricant	Osmolality(mmol/kg)		pH	Viscositycps, 10 rpm			CC_50_	ED_50_		Therapeutic index		Listed ingredients^5^
				@ 25°C	@ 37°C							
*Aqueous-based*												
Astroglide		6113	4.0	207	181		0.9	0.8		1		Glycerin, Polyethylene glycol, Polyquaternium, Methylparaben, Propylparaben
Elbow Grease		3865	5.7	3159	808		5.7	0.4		14		Glycerin, Glycereth, Hydroxyethyl cellulose, Methylparaben, Propylparaben, Imidazolidinyl Urea
Good Clean Love		269	4.8	3003	3179		>1000	0.6		>1000		Xanthan gum, Agar, organic Aloe barbendensis leaf juice, Lemon extract, Vanilla extract, Potassium sorbate, Benzoic acid
Gynol II^1^		1406	4.7	22783^3^	15970^3^		0.4	0.1		4		Nonoxynol-9 (2%), Lactic acid, Methylparaben, Povidone, Propylene glycol, Sodium carboxymethyl cellulose, Sorbic acid, Sorbitol solution
ID Glide Ultra long-lasting		3150	5.2	751	601		4.8	0.8		6		Glycerin, Polyethylene glycol, Cellulose polymer, Polyethylene oxide, Sodium benzoate, Methylparaben, Carbomer 981, Tetrahydroxypropyl ethylenediamine, Diazolidinyl Urea, EDTA
KY Jelly		2510	4.5	5913	6560		11.8	5		2		Chlorhexidine gluconate, Gluconolactone, Glycerin, Hydroxyethyl cellulose, Methylparaben, NaOH
PRÉ		502	7.3	1683	1051		308	4.4		70		Hydroxyethyl cellulose, Pluronic 127, NaCl, NaH_2_PO_4_, Carbomer 934, Methylparaben, NaOH, Arabinogalactan, KH_2_PO_4_, Propylparaben, Potassium phosphate
Replens		1875	2.9	3935	4018		19.8	0.5		40		Glycerin, Mineral oil, Polycarbophil Carbomer homopolymer type B, Hydrogenated palm oil glyceride, Sorbic acid, Methylparaben, Sodium hydroxide
Slippery Stuff		26	6.8	3640	3744		>1000	26		>40		Polyethylene oxide, Sodium carbomer, Methylparaben
Sliquid Organic		106	6.8	534	725		3.0	3.9		0.8		Plant cellulose (from cotton), Aloe barbadensis, Tocopherols (vitamin E), Cyamopsis (guar conditioners), Hibiscus extract, Flax extract, Green tea extract, Sunflower seed extract, Citric acid, Phenoxyethanol (rose ether)
*Lipid-based*												
Boy Butter H_2_O		1307	7.4	1558	853	ND^4^			ND		ND	Carbomer, Tocopheryl acetate, Methylparaben, Propylparaben, Propylene glycol, Glycerin, PEG-100 stearate, Glyceryl Stearate, Tricaprylin, Shea butter, Cyclopentasiloane, Aloe Vera extract, Vanilla extract, Diazolidinyl urea, Yellow #5, Triethanolamine
Boy Butter original		NA^2^	NA	4840	2600	ND			ND		ND	Partially hydrogenated vegetable oil, Glycerin, Polysorbate 60, Tocoheryl acetate, Glyceryl stearate, Phenyl trimethicone
*Silicone-based*												
FC 2 lubricant		NA	NA	330	330	ND			ND		ND	Polydimethyl siloxane
Wet Platinum		NA	NA	145	113	ND			ND		ND	Dimethicone, Cyclomethicone, Dimethiconol

1 Gynol II is a spermicidal gel which contains 2% nonoxynol-9.

2 NA = not applicable.

3 Viscosity was determined at 5 rpm to bring torque values into a reliable range.

4 ND = not determined.

5 Ingredient listed in the order it appears on the package.

### Lubricant Safety Testing

**Figure 1:**
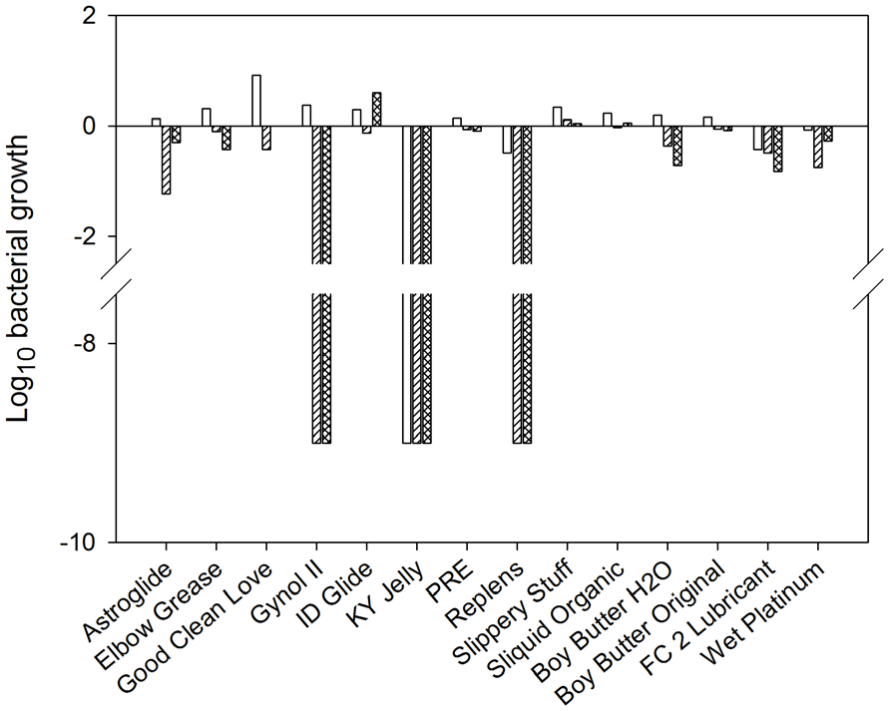
Impact of the over-the-counter lubricants on *Lactobacillus species* viability. *Lactobacillus* species (*L. crispatus* (open bar); *L. jensenii* 25258 (diagonal line bar); *L. jensenii* 28Ab (diamond hatch bar)) were cultured in the presence of lubricants for 30 min then plated. The reduction of colony forming units was compared to control cultures. The data are presented as the Log_10_ growth compared to the control cultures.

*Lactobacillus* species are important for the maintenance of the female genital tract as they produce lactic acid and hydrogen peroxide. Loss of lactobacilli often coincides with an increased susceptibility to HIV-1 infection [31]. Therefore, three species of lactobacilli were tested for retention of viability in the presence of the lubricants (Figure 1). A 1 log_10_ reduction in bacterial growth was considered a significant loss. Astroglide reduced the growth of one of the *L. jensenii* by 1.2 log_10_ while it had no effect on the other two lactobacilli strains. Replens and Gynol II showed complete loss of both *L. jensenii* strains while the viability of *L. crispatus* was not affected by either product. The *L. jensenii* appear to be more sensitive to N9 than the *L. crispatus*. However, it is not clear why the *L. jensenii* were sensitive to Replens. Exposure to KY Jelly resulted in complete loss of the three strains of lactobacilli, likely attributed to chlorhexidine in the formulation which is a known bactericidal compound. The remaining aqueous-, lipid-, and silicone-based products had no detrimental effect on bacterial viability.

**Figure 2:**
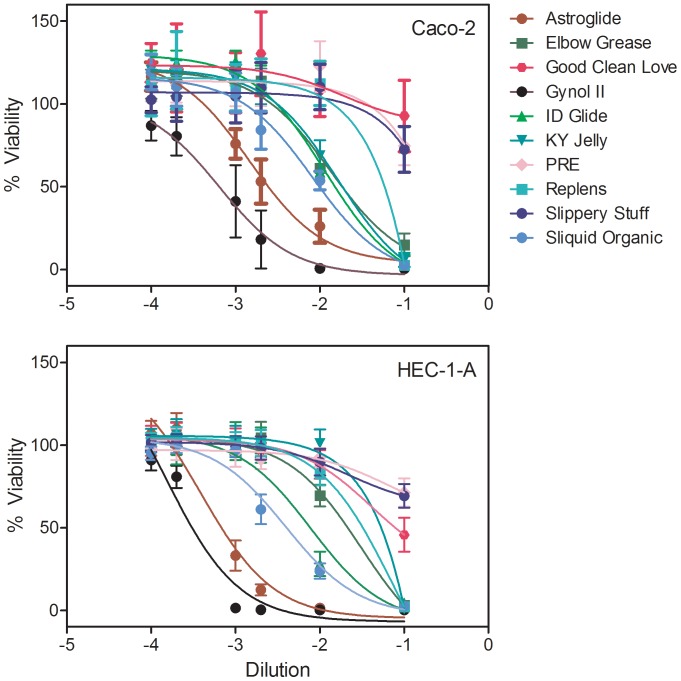
Impact of the over-the-counter aqueous-based lubricants on epithelial cell line viability. Caco-2 (upper panel) and HEC-1-A (lower panel) epithelial cells were treated with serial dilutions of the indicated lubricants for 24 h and their viability was measured and presented as the %Viability of the control (untreated) cells. The data presented are the mean ± standard deviation of 5 independent experiments.

**Figure 3:**
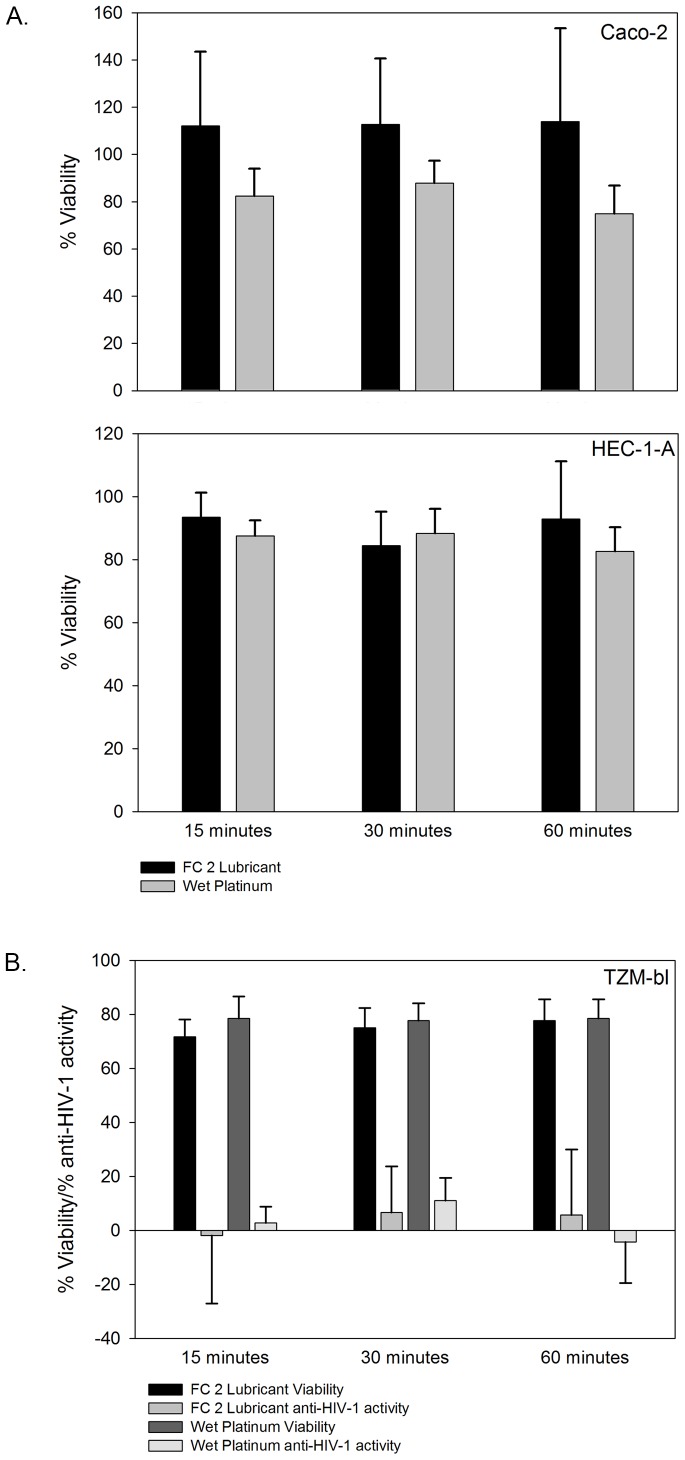
Impact of the over-the-counter silicone-based lubricants on epithelial cell lines. (A) Caco-2 and HEC-1-A epithelial cell lines were treated with Female Condom 2 (FC 2) lubricant or Wet Platinum for 15, 30 or 60 min and %Viability of the control (untreated) cells was measured. (B) TZM-bl cells were treated with FC2 lubricant or Wet Platinum for 15, 30, or 60 min and %Viability or %Anti-HIV-1 activity of the control (untreated) cells was measured. The data presented are the mean ± standard deviation of 5 independent experiments.

Because the products will interact directly with epithelial cells, dilutions of aqueous-based products were made in the appropriate medium for safety testing on Caco-2 and HEC-1-A epithelial cell lines (Figure 2). Gynol II demonstrated significant (p<0.0004) toxicity to the epithelial cells with a ≥1∶1000 dilution needed to maintain culture viability. Treatment with Astroglide was similar to the Gynol II curve with a ≥1∶1000 required to maintain culture viability. Conversely the viability curves for Elbow Grease, ID Glide, KY Jelly, Replens, and Sliquid Organic were similar in that each product required a ≥1∶100 dilution to maintain culture viability. Minimal impact on culture viability was associated with Good Clean Love, PRÉ, and Slippery Stuff products. In general, the loss of cell viability correlated to the osmolality of the gels; the higher the solute concentration, the greater the dilution that was needed to maintain viability. Interestingly, Slippery Stuff was the most hypo-osmolar gel tested, but retained cell viability. Because FC 2 lubricant and Wet Platinum are silicone-based gels and not water soluble, they cannot be diluted appropriately in culture medium. To address this issue, the silicone-based products were applied directly to the cells for 15, 30, or 60 min before medium was applied. Exposures up to 60 min modestly affect the viability of the epithelial cell lines (Figure 3). Due to the physical nature of the lipid-based products, they could not reliably be tested for their impact on epithelial cell line viability.

**Figure 4:**
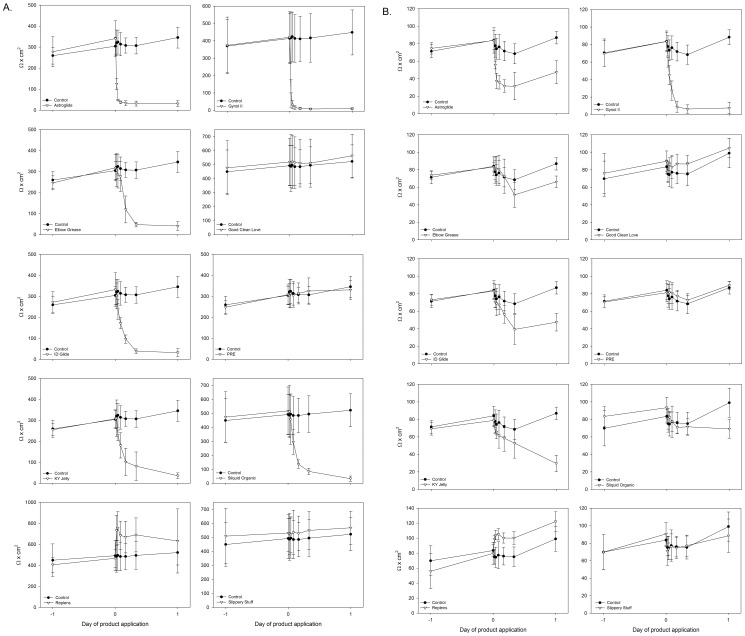
Effect of the over-the-counter aqueous-based lubricants on epithelial cell line monolayer integrity. (A) Caco-2 or (B) HEC-1-A epithelial cell lines were grown on transwell supports until a polarized monolayer was established. A 1∶10 dilution of each of the lubricants was applied to the apical surface to allow for even spread over the cell surface, and the monolayers were followed over a 24 h period. The data presented are the mean ± standard deviation of 5 independent experiments.

Hyperosmolar products lead to epithelial disruption [32], [33]. This is important because the mucosal epithelium is a functional barrier against potential pathogens [34], [35]. To measure changes in epithelial monolayer integrity, Caco-2 and HEC-1-A cell lines were grown on transwell supports and allowed to form monolayers. The integrity of the monolayer was measured using a volt meter (for aqueous-based gels) or by transmission of fluorescent beads which are similar in size to HIV-1 (for silicone-based liquids). Because of the physical attributes of the lipid-based gels, their impact on epithelial monolayers could not be tested. For the aqueous gels, the transepithelial resistance (TER) of the untreated monolayer varied by <10% over the 3 day testing period. Minimal dilutions of the aqueous-based gels were made for even spread across the cells and applied to the apical surface. The addition of Gynol II to Caco-2 or HEC-1-A monolayers caused significant reductions (75% or 21%; p≤0.0012) in their TER by 30 min which continued to decrease until it reached background TER levels by 4 h (Figure 4A & 4B). These data are consistent with our previous findings for gels containing nonoxynol-9 [21], [27]. In general, the addition of the hyperosmolar gels resulted in significant (p<0.0004) loss of both Caco-2 and HEC-1-A TER by 30 min to 1 h after application. Caco-2 cells (Figure 4A) showed more dramatic and sustained loss of TER as compared to the HEC-1-A cells (Figure 4B). PRÉ and Slippery Stuff did not affect the Caco-2 or HEC-1-A epithelial cell TER as the treated well measurements virtually overlapped the control well measurements. While not affecting the Caco-2 TER, Good Clean Love did modestly increase the TER of the HEC-1-A cells. These three gels also retained epithelial viability (Figure 2). Interestingly, Sliquid Organic, a slightly hypo-osmolar gel, impacted the epithelial cell TER in a similar manner as the hyperosmolar gels; Caco-2 cells had a significant loss of the monolayer by 2 h (p<0.0004) and HEC-1-A cells had a significant loss at 24 h (p<0.0004). Replens was the only product to reproducibly increase the TER for both Caco-2 and HEC-1-A cells (p<0.0004).

**Figure 5:**
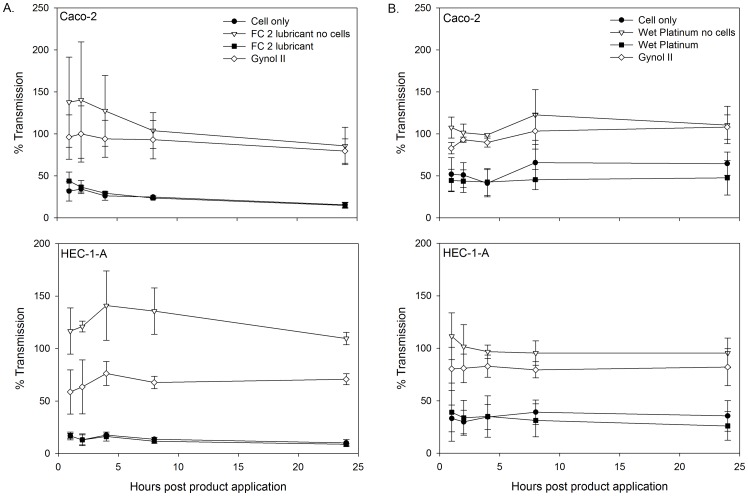
Effect of the over-the-counter silicone-based lubricants on epithelial cell line monolayer integrity. (A) Female Condom 2 lubricant (FC2) or (B) Wet Platinum were evaluated for their impact on Caco-2 and HEC-1-A epithelial cell line monolayers. Lubricant was directly applied to the apical surface of the monolayers for 60 min and then medium containing fluorescent microbeads was applied. Baselateral supernatant was collected over a 24 h period and the fluorescence was measured. The data presented are the %Transmission and represents the mean ± standard deviation of 5 independent experiments.

Because the silicone-based products would coat the electrodes of the volt meter and result in inaccurate readings, their effect was measured as % transmission of fluorescent microbeads through stable epithelial cell monolayers. Over the course of 24 h, the % transmission of the beads through Caco-2 and HEC-1-A cell monolayers did not significantly differ between cell only wells and cells treated with FC 2 lubricant or Wet Platinum (Figure 5). This was not due to the silicone lubricants preventing the microbeads from passing through the membrane because wells with either lubricant alone (with no cells) did not impede bead transmission through the filter as the % transmission was ≥100% throughout the 24 h. As controls, wells with Gynol II, an aqueous-based product which induced a loss of the monolayer (Figure 4A & 4B), were included and showed significant transmission (p<0.0008) of the beads by 1 h for HEC-1-A cells and by 2 h for Caco-2 cells (Figure 5). These data demonstrate silicone-based products do not affect the epithelial monolayer integrity while Gynol II that caused a loss of epithelial integrity allowed passage of the microbeads.

**Figure 6:**
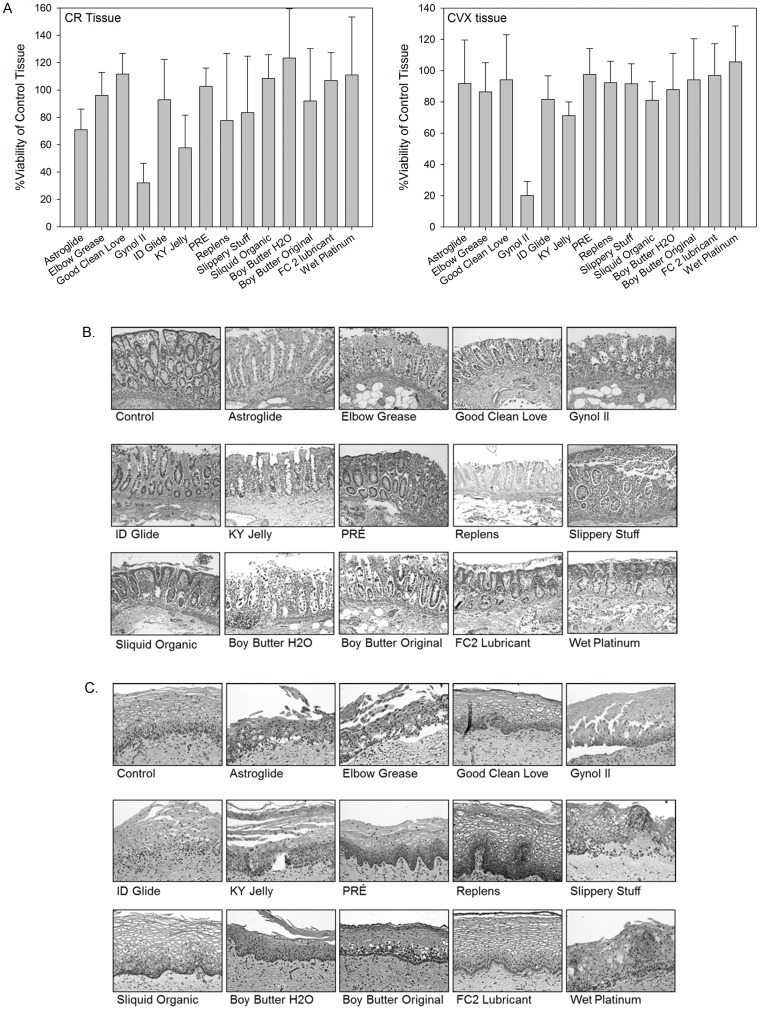
Impact of the over-the-counter lubricants on colorectal (CR) and ectocervical (CVX) tissue viability and architecture. *Ex vivo* tissue was placed in transwell supports with the luminal surface exposed to the air. The edges were sealed to ensure the lubricant was exposed to the luminal epithelium in duplicate cultures. After an overnight exposure, tissue was washed with one piece further cultured in medium containing 1-(4,5-dimethylthiazol-2-yl)-3,5-diphenylformazan for the MTT assay (A) or the other piece fixed in paraformaldehyde for hematoxylin and eosin staining of CR tissue (B) and CVX tissue (C). The MTT assay represents the mean ± standard deviation of a minimum of 5 independent tissues. The histology is representative of one of those tissues.

To further assess lubricant safety, undiluted products were applied to the apical surfaces of polarized colorectal and ectocervical explant cultures and viability was measured using the MTT assay and histology. Viability of the ectocervical and colorectal explants was retained for most products with the exception of Gynol II (20% and 33% viability, respectively; p<0.0018), Astroglide (92% and 71% viability, respectively; p<0.0018), and KY Jelly (71% and 58% viability, respectively; p<0.0018) (Figure 6A). Gynol II contains 2% nonoxynol-9 which has shown to be toxic to cells and tissues [21], [28], [29], [36]. The osmolality of Astroglide likely influenced the viability of the mucosal tissue. The significant loss of tissue viability by KY Jelly was not anticipated; however, the extended exposure to chlorhexidine may have affected tissue viability. Elbow Grease, ID Glide, and Sliquid Organic significantly (p<0.0018) reduced the viability of ectocervical explants, but did not affect colorectal explants viability. Conversely, Slippery Stuff significantly (p<0.0018) reduced the viability of colorectal tissue, but had no effect on ectocervical tissue viability.

Histological evaluation showed stripping/fracture of the colorectal and ectocervical epithelium by most of the hyperosmolar products (Figure 6B & 6C). Replens stripped the colorectal epithelium but showed no damage to the ectocervical epithelium. The products that were nearly iso-osmolar (Good Clean Love and PRÉ) did not show loss of viability or epithelium. The hypo-osmolar lubricant Slippery Stuff did show loss of colorectal but not ectocervical epithelium while Sliquid Organic did not affect either tissue epithelium. Of both lipid-based lubricants, Boy Butter H_2_O reduced the viability of the ectocervical tissue, but did not affect the colorectal tissue and caused stripping of the colorectal epithelium and little damage to the ectocervical epithelium. Boy Butter Original also showed some stripping of the colorectal epithelium but no damage to the ectocervical tissue. The silicone-based products showed no effect on tissue viability or the epithelium. Collectively, the nearly iso-osmolar and silicone-based lubricants were the safest for epithelial cells and mucosal tissues.

### Lubricant Anti-HIV-1 Activity

Previous studies suggested lubricant products have anti-HIV-1 activity which could be attributed to some of their constituents [37], [38]. However, there was no correlation to the toxicity associated to these products in these studies. To investigate this, dilutions of each of the aqueous-based products were applied to TZM-bl cells alone or with HIV-1_BaL_ to determine the CC_50_ and the ED_50_, respectively, to calculate the therapeutic index. The majority of the products tested showed negligible therapeutic indices (<20) (Table 1). There were modest therapeutic indices associated with PRÉ, Replens and Slippery Stuff and a high therapeutic index associated with Good Clean Love. None of the ingredients listed for these four products would be suggestive of higher therapeutic indices noted by the TZM-bl assay (Table 1). Interestingly, Sliquid Organic, which contains green tea extract, did not exhibit anti-HIV-1 activity beyond it cytotoxic concentration in the TZM-bl assay. Likewise, Gynol II which contains nonoxynol-9 exhibited no therapeutic activity due to high levels of toxicity. Because the silicone-based lubricants, FC 2 lubricant and Wet Platinum could not be diluted in aqueous medium, we investigated their inhibitory activity by their length of time exposed to the TZM-bl cells. When cultured with TZM-bl cells, no anti-HIV-1 activity was noted (Figure 3B).

### Effect of Lubricants on HIV-1 Infection of Mucosal Tissue

**Figure 7:**
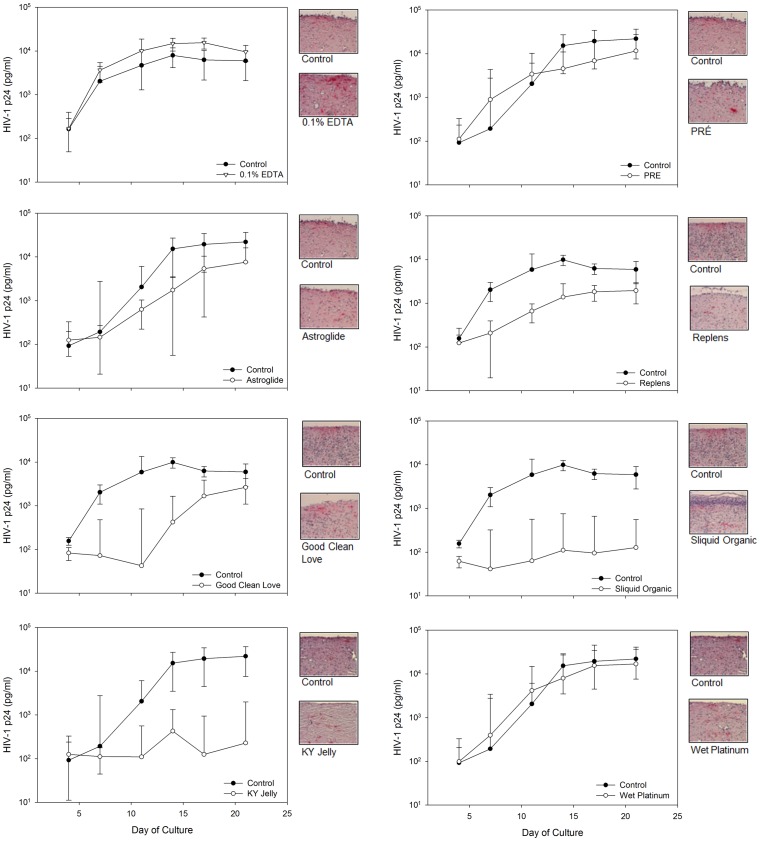
Effect lubricants have on HIV-1 infection of ectocervical tissue. Ectocervical tissue was placed in transwell supports with the luminal surface exposed to the air. The edges were sealed to ensure the lubricant was exposed to the luminal epithelium in duplicate cultures. Tissues were cultured with the indicated lubricant overnight. Controls consisted of no treatment or additional well reserved for 0.1% EDTA. After washing, the control tissues and those exposed to lubricants were rested for 2 h while the reserved tissue was treated with 0.1% EDTA during the 2 h. The EDTA-treated tissues were then washed and all tissues were exposed to HIV-1 overnight. The tissues were washed and cultured for 21 days; the medium in the basolateral chamber was replenished every 3 to 4 days. HIV-1 replication was monitored in saved supernatant by p24 ELISA. At study end, tissue was fixed and stained for HIV-1 infected cells by immunohistochemistry. The data shown represent the median ± the 95% confidence interval of 4 to 5 independent tissues. The immunohistochemistry is representative of one of those tissues.

The combined toxicity data and lack of associated anti-HIV-1 activity for most products suggest alterations or removal of the epithelial barrier could increase the susceptibility of the tissue to HIV-1 infection. To test this hypothesis, polarized explants were set-up and exposed to selected lubricant products or 0.1% EDTA. After exposure, the explants were washed and then exposed to HIV-1. The products chosen were Astroglide (the most hyperosmolar gel), Good Clean Love (iso-osmolar gel with a high therapeutic index), KY Jelly (moderately hyperosmolar and widely available OTC lubricant), Replens (moderately hyperosmolar gel), Sliquid Organic (slightly hypo-osmolar gel and contains green tea extract), PRÉ (nearly iso-osmolar gel with a modest therapeutic index), and Wet Platinum (silicone-based gel). EDTA was chosen as a positive control for these studies because it increased susceptibility of mice to HSV-2 infection [39]. Furthermore, given its strong chelating ability it complexes calcium which influences tight junctions by opening desmosomes in the epithelium causing enhanced permeability [40], [41], [42]. Ectocervical explants exposed to 0.1% EDTA replicated HIV-1 to slightly elevated levels as compared to the controls, but this was a significant increase in virus output (p<0.0001) (Figure 7). None of the lubricants tested demonstrated a similar increase in HIV-1 replication. While Astroglide (p = 0.0002), Good Clean Love (p = 0.01, not significant after adjustment for multiple comparisons), and Replens (p = 0.0001) showed a delay in HIV-1 replication with lower peak p24 levels as compared to the control explants, all explants were infected as confirmed by IHC (Figure 7). Explants treated with PRÉ and Wet Platinum showed similar HIV-1 replication kinetics to the control explants and all explants were infected by IHC. KY Jelly-treated explants did not replicate HIV-1 and only 2 of 10 explants showed HIV-1 infected cells by IHC. Sliquid Organic reduced HIV-1 infection by 1.5 log_10_ and half of the explants demonstrated infection by IHC (Figure 7). This is in contrast to the TZM-bl assay showing no therapeutic benefit and suggests that green tea has a very modest impact on HIV-1 infection in this model. When considered with the tissue toxicity data (Table 1), it would suggest the loss of viable cells in the explants treated with KY Jelly and Sliquid Organic resulted in the absence of HIV-1 infection.

## Discussion

Lubricants are commonly used to enhance pleasure while possibly reducing trauma during coitus. For example, while dyspareunia increases with the age of a woman, up to 46% of women between 18 to 45 years old experience it at some point [43]. Clinicians often prescribe the use of OTC lubricants to alleviate its symptoms. Likewise, receptive anal intercourse can lead to trauma of the anus/rectum. Lubricants could reduce this trauma and are used by a significant number of men engaging in receptive anal intercourse [19]. However, a comprehensive analysis of the safety of lubricant products has not been done. Our findings show that six of the 10 aqueous-based lubricants tested were hyperosmolar and reflected the cellular toxicity and damage to epithelial monolayers and explant epithelium. Two nearly iso-osmolar gels and both silicone-based gels evaluated here showed no cellular toxicity or damage to epithelial monolayers and explant epithelium. Despite the lack of increased HIV-1 infection in ectocervical tissue, these results convey the importance of using condoms in conjunction with compatible lubricants.

Preservatives are added to OTC lubricant products to ensure product shelf-life. The choice of preservative could have an impact on the microflora of the genital and GI tracts. Chlorhexidine gluconate is a bactericidal compound frequently used in pre-operative skin preparations and mouth washes for gingivitis [44], [45]. KY Jelly contains chlorhexidine gluconate and was the only lubricant that completely killed the *Lactobacillus* species tested. Methyl- and propylparaben are bactericidal preservatives commonly used in cosmetics and pharmaceutical preparations. These preservatives are added to topical microbicide gel formulations to protect from microbial contamination. Nine of the 11 OTC aqueous-containing products tested contained methylparaben and/or propylparaben. However, only Gynol II and Replens significantly reduced the viability of two of the three lactobacillus species tested. It’s unknown why the other OTC lubricant products that contain these preservatives did not affect the *Lactobacillus* viability, but it could be due to concentrations of the parabens used in those gels. Additionally, Gynol II contains nonoxynol-9 which has a detrimental effect on *Lactobacillus* viability in women who use nonoxynol-9-containing gels [46], [47]. Collectively, changes in vaginal flora and in particular loss of *Lactobacillus* species are associated with the development of bacterial vaginosis (BV) [31]. KY Jelly has been used in a number of microbicide safety trials as a “placebo”. Overall, no significant increase in BV was observed in these trials. However, several women using KY Jelly developed BV during the study as compared to none in the active gel arms [5], [6], [7], [9], [10]. These trials did not include a “no gel” arm that would have been helpful in defining BV incidence in the population. Our data would suggest consistent vaginal use of Gynol II and KY Jelly could lead to changes in lactobacilli populations. However, more research is needed to define the role of extended OTC lubricant product use and incident BV.

Previous testing using several *in vitro* [48], [49], [50] and *ex vivo* [51] models showed some aqueous-based lubricants reduced epithelial cell viability. Our evaluation of lubricants on epithelial cell lines in general showed decreasing cell viability associated with increasing osmolality of the gels. The hyperosmolar lubricants also perturbed epithelial monolayers showing a delayed reduction of the TER in the HEC-1-A cell line and a swift reduction of TER in the Caco-2 cell line. Interestingly, the slightly hypo-osmolar gel, Sliquid Organic, showed similar epithelial toxicity and loss of monolayer retention to the hyperosmolar gels. The reasons for this are not clear as there were no obvious ingredients on the package insert (Table 1) which would indicate toxicity. The remaining near iso-osmolar and hypo-osmolar gels did not affect the epithelial cell viability or monolayers. Of interest, the silicone-based lubricants showed no effect on the epithelial viability or monolayer using our testing methods. The effects to the epithelial monolayers were predictive of the epithelium retention on the polarized colorectal and ectocervical tissues. Three products showed a consistent decrease in tissue viability: Astroglide, Gynol II and KY Jelly. Astroglide’s osmolality likely impacted the tissue viability while chlorhexidine affected tissue viability in KY Jelly exposed tissue. Gynol II contains 2% N9 which decrease cellular/tissue viability *ex vivo* [36], [52]. In this study, a 24 h exposure to Gynol II reduced tissue viability by 67% to 80%. When viewed histologically, the ectocervical tissues showed fractured epithelium and were acellular while the colorectal tissues were stripped of their epithelium and were necrotic. The impact of the hyperosmolar and nonoxynol-9-containing gels on colorectal tissue was not unexpected as clinical data has shown stripping of the epithelium [32], [53]. These studies followed patients closely (within 30 min of dosing) and obtained biopsies to perform histological evaluation. Epithelial findings of the ectocervix/vagina have not been documented after vaginal use of these products. This is likely due to inconsistent observations after the last vaginal dose of gel and the lack of biopsies taken for histological testing. However, the stratified squamous epithelium of the ectocervix/vagina may not show the apparent epithelial disruption as does the columnar epithelium of the gastrointestinal tract. The other hyper- and hypo-osmolar gels had modest reductions of tissue viability with commensurate changes in mucosal epithelium. The only exception was Sliquid Organic which showed modest loss of ectocervical tissue viability and good epithelial retention of both tissues. Good Clean Love and PRÉ – iso-osmolar gels – retained tissue viability and epithelium. The silicone-based lubricants showed no alteration of tissue viability or epithelium either. Interestingly, while marketed specifically for rectal use, both Boy Butter products showed epithelial striping of the colorectal tissue with only modest changes seen in ectocervical tissue. These are both lipid-based lubricants and not intended for use with condoms. Overall, the safest lubricants, based on the testing presented here, appear to be the nearly iso-osmolar gels and silicone-based liquids.

OTC lubricants typically do not contain drugs or pharmacologically active agents known to have activity against HIV-1 (Table 1). However, they do contain a number of ingredients that contribute to the physical characteristics (viscosity, lubrication) and stabilization (antioxidants, preservatives). Two such agents are glycerin and EDTA [39]. Previous work suggested that OTC lubricant products, in particular Astroglide, may have anti-HIV-1 activity based on activity afforded by inactive or excipients ingredients present in the product [37], [38]. Astroglide was shown to block cell-associated or cell-free HIV-1 infection [37]. Further work showed glycerol and polyquaternium-32 were the active anti-HIV-1 compounds in Astroglide [38]. However, none of their work correlated the anti-viral activity to the cellular toxicity associated with the lubricants. We show here the majority of the lubricants did not possess any anti-HIV-1 activity beyond their toxic concentrations. This was corroborated by a recent paper showing the same four OTC lubricants tested in this paper along with other iterations of them had no anti-HIV-1 activity [48]. However, it was suggested by Begay et al. [48] that the polyquaternium-32 could increase HIV-1 infection. Not being able to source the polyquaternium-32, a similar molecule (MADQUAT) was used in their assay. They found that MADQUAT increased HIV-1 infection. The results presented here show that in *ex vivo* mucosal tissue, Astroglide did not increase HIV-1 infection. Additionally, the three aqueous-based lubricants that had minimal loss of viability, Good Clean Love, PRÉ, and Slippery Stuff, demonstrated modest anti-HIV-1 activity using our *in vitro* assay. However, no reduction of HIV-1 infection was noted in ectocervical tissue by the two lubricants tested. This could be an artifact of the *in vitro* assay because the viscosity of these gels could reduce the infection of the indicator cell line. It should be noted when we evaluated the hydroxyethyl cellulose (HEC) gel – the universal placebo gel – with similar viscosity, no anti-HIV-1 activity was noted [27], . The HEC gel contains 2.7% HEC polymer with other salts [54] while the other gels use agar, xanthan gum, and carbomer at unknown concentrations (Table 1). These other gelling agents could impact HIV-1 infection of the TZM-bl cells at high concentrations. Sliquid Organic, which contains green tea extract and showed no *in vitro* anti-HIV-1 activity, did provide modest protection against HIV-1 infection in the ectocervical tissue. The active ingredient in green tea, epigallocatechin gallate, has been shown to have anti-HIV-1 [55] and HSV [56] properties *in vitro* likely by interfering with viral attachment to its cellular targets. However, the effectiveness of this extract against HIV-1 infection in a clinical setting would likely be poor. Proven effective anti-virals such as PRO 2000 [57], [58] and tenofovir [59], [60] when used as a topical microbicide have provided no to modest protection against HIV-1 acquisition in high-risk women. Consequently, the benefit of using a lubricant for HIV-1 prevention is limited.

The mechanisms of HIV-1 transmission during coitus have not been fully elucidated, but the epithelium is an important barrier to block entry [34], [35]. OTC spermicides and first generation, HIV-1 entry inhibitor microbicide gels were shown to increase susceptibility to HIV-1 infection in clinical trials [61], [62]. Subsequent analysis suggested that epithelial disruption might be a primary reason for their failure. Nonoxynol-9 and cellulose sulfate gels disrupt epithelial monolayers *in vitro* [27], [63] which can result in increased transmission of HIV-1 across the breached epithelium [63], [64]. Astroglide, KY Jelly, and other hyperosmolar lubricant products described here and elsewhere [48], [49], [65], [66], [67] show striping/facture of the epithelium with no anti-HIV-1 activity. Indeed, the HSV/mouse models suggest that these products may increase susceptibility to infection [39], [65], [66], [67]. Glycerin and EDTA were shown to be associated with the increased HSV infection in the mice. The glycerin likely removes the epithelium through osmotic changes while EDTA chelates the calcium and magnesium ions that are important for tight junction formation between epithelial cells and opens up the epithelium. We treated ectocervical tissue with 0.1% EDTA for 2 h and then exposed to HIV-1. We found a modest, but significant, increase in HIV-1 replication in the EDTA-exposed tissue as compared to the untreated tissue. Despite showing epithelial damage, the lubricant treated tissues did not increase HIV-1 replication. These data are supported by a paper presented at Microbicides 2008 showing little changes in gastrointestinal tract permeability after taking 20 pinch biopsies from the mucosa as compared to exposure to nonoxynol-9 [68]. Participants who had biopsies taken had similar plasma levels of a radiolabeled isotope, ^99m^Tc-DTPA, as compared to participants who received an enema containing a saline derivative. Conversely, participants who received an enema containing nonoxynol-9 had 22-fold more radiolabeled isotope in their plasma than the saline derivative. Collectively, these data suggest that epithelial trauma alone may not be sufficient to increase HIV-1 infection, but other factors such as an inflammatory milieu may also be necessary.

There are several limitations to our evaluation of OTC lubricants. The majority of our testing was restricted to aqueous-based gels and silicone-based liquids. We attempted to evaluate lipid-based emulsions in our assays and have not had satisfactory results. For example, Boy Butter Original and Boy Butter H_2_O are popular lubricants used by MSM. However, their cream-like consistency does not allow them to be utilized in our assays due to our inability to wash the lubricant from the cells. This limits their thorough evaluation. However, we were able to evaluate their safety on *ex vivo* tissue directly. Also, this is the first time *ex vivo* tissue has been used to model susceptibility to HIV-1. Using EDTA as the control to increase susceptibility, a modest increase in HIV-1 replication was noted despite more p24 expressing cells consistently observed by IHC. *Ex vivo* tissue is not necessarily representative of the *in vivo* environment because of lack of tissue regeneration, lack of recruitment of immune cells, and independence from hormones.

While these data are compelling, additional research is needed to determine if hyperosmolar lubricants cause sub-clinical damage to the mucosal epithelium and lamina propria which could potentially lead to increased susceptibility to HIV-1 and other sexually transmitted diseases. Indeed, a recent paper evaluating persons who consistently use lubricants during RAI showed a higher incidence of sexually transmitted diseases [69]. However, the type of lubricant (e.g. aqueous-based versus silicon-based) was not defined in this study. Taken together, condoms, irrespective of male or female, remain the best available method to prevent the acquisition of HIV-1 and sexually transmitted diseases and should be used with compatible lubricants.
